# Timing of surgery in acute deep partial-thickness burns: A study protocol

**DOI:** 10.1371/journal.pone.0299809

**Published:** 2024-03-11

**Authors:** Roos F. C. Salemans, Denise van Uden, Margriet E. van Baar, Tjitske M. Haanstra, Carine H. M. van Schie, Paul P. M. van Zuijlen, Ymke Lucas, Sonja M. H. J. Scholten-Jaegers, Annebeth Meij-de Vries, Fiona M. Wood, Dale W. Edgar, Inge Spronk, Cornelis H. van der Vlies, National Burn Care, Education & Research group, the Netherlands

**Affiliations:** 1 Trauma Research Unit Department of Surgery, Erasmus MC, University Medical Centre Rotterdam, Rotterdam, the Netherlands; 2 Burn Centre, Maasstad Hospital, Rotterdam, the Netherlands; 3 Association of Dutch Burn Centres, Maasstad Hospital, Rotterdam, the Netherlands; 4 Department of Public Health, Erasmus MC, University Medical Centre Rotterdam, Rotterdam, the Netherlands; 5 Department of Dermal Therapy, Faculty of Health, Nutrition & Sport, The Hague University of Applied Sciences, The Hague, the Netherlands; 6 Dutch Burns Foundation, Beverwijk, the Netherlands; 7 Research Group Relational Care, Centre of Expertise Health Innovation, The Hague University of Applied Sciences, The Hague, the Netherlands; 8 Burn Centre, Red Cross Hospital, Beverwijk, the Netherlands; 9 Department of Plastic and Reconstructive Surgery, Red Cross Hospital, Beverwijk, the Netherlands; 10 Department of Plastic Reconstructive and Hand Surgery, Amsterdam Movement Sciences Institute, Amsterdam UMC, Location VUmc, Amsterdam, the Netherlands; 11 Paediatric Surgical Centre, Emma’s Children’s Hospital, Amsterdam UMC, Location AMC, Amsterdam, the Netherlands; 12 Burn Centre, Martini Hospital, Groningen, the Netherlands; 13 Department of Surgery, Red Cross Hospital, Beverwijk, the Netherlands; 14 Fiona Wood Foundation, Fiona Stanley Hospital, Murdoch, Western Australia, Australia; 15 State Adult Burn Unit, Fiona Stanley Hospital, South Metropolitan Health Service, Murdoch, Western Australia, Australia; 16 Institute for Health Research, Burn Injury Research Node, The University of Notre Dame Australia, Fremantle, Western Australia, Australia; 17 Burn Injury Research Unit, Faculty of Medicine and Dentistry, University of Western Australia, Crawley, Western Australia, Australia; 18 Safety and Quality Unit, Armadale Kalamunda Group Health Service, East Metropolitan Health Service, Mt Nasura, Western Australia, Australia; 19 Department of Trauma and Burn Surgery, Maasstad Hospital, Rotterdam, the Netherlands; University of California Davis, UNITED STATES

## Abstract

For deep partial-thickness burns no consensus on the optimal treatment has been reached due to conflicting study outcomes with low quality evidence. Treatment options in high- and middle-income countries include conservative treatment with delayed excision and grafting if needed; and early excision and grafting. The majority of timing of surgery studies focus on survival rather than on quality of life. This study protocol describes a study that aims to compare long-term scar quality, clinical outcomes, and patient-reported outcomes between the treatment options. A multicentre prospective study will be conducted in the three Dutch burn centres (Rotterdam, Beverwijk, and Groningen). All adult patients with acute deep-partial thickness burns, based on healing potential with Laser Doppler Imaging, are eligible for inclusion. During a nine-month baseline period, standard practice will be monitored. This includes conservative treatment with dressings and topical agents, and excision and grafting of residual defects if needed 14–21 days post-burn. The subsequent nine months, early surgery is advocated, involving excision and grafting in the first week to ten days post-burn. The primary outcome compared between the two groups is long-term scar quality assessed by the Patient and Observer Scar Assessment Scale 3.0 twelve months after discharge. Secondary outcomes include clinical outcomes and patient-reported outcomes like quality of life and return to work. The aim of the study is to assess long-term scar quality in deep partial-thickness burns after conservative treatment with delayed excision and grafting if needed, compared to early excision and grafting. Adding to the ongoing debate on the optimal treatment of these burns. The broad range of studied outcomes will be used for the development of a decision aid for deep partial-thickness burns, to fully inform patients at the point of consent to surgery and support optimal person-centred care.

## Introduction

Burns are one of the most common types of trauma worldwide, causing significant disability [1, 2]. Today, acute burn care in high- and middle-income countries has improved to the point where the focus has shifted from survival as main outcome to outcomes like quality of life, leaving room to broaden our view to other challenges [3]. One of these challenges is the timing of surgery. The severity of a burn, determined primarily by total body surface area (TBSA) and depth of the injury, influences reparative healing potential, and is a primary indicator of the need for surgery [4–6]. For deep partial-thickness burns, currently no consensus regarding the optimal treatment has been reached, which remains a topic of debate [7]. Contrary to superficial partial-thickness burns and full-thickness burns, where the optimal treatment is evident. Superficial partial-thickness burns affect the epidermal and upper dermal layers of the skin, heal within 14 days and do not require surgery [8]. Full-thickness burns fully penetrate all layers of the skin into the subcutis. No spontaneous healing is expected and excision and grafting are always indicated [8–11].

The healing potential of deep partial-thickness burns varies with the longer time to healing being associated with increased scar risk. Two main treatment strategies are currently applied in high- and middle-income countries: 1) conservative treatment with delayed excision and grafting if needed; 2) early excision and grafting. Conservative treatment involves applying dressings and topical antimicrobial and antiseptic agents to the burn, after which spontaneous healing is awaited. If the remaining defects do not heal spontaneously within 14 to 21 days, the burn specialist can decide to perform excision and grafting. In case of early surgery, excision and grafting of the burn is performed within the first week to ten days post-burn [9].

Both treatment strategies have advantages and disadvantages. Early excision and grafting of deep partial-thickness burns is recommended by the International Society for Burn Injury for faster recovery, less pain, and possibly improved long-term scar outcomes [9]. Conservative treatment, on the other hand, avoids the mental and physical stress that can be associated with surgery, particularly for the frail or anxious patient [12]. Moreover, conservative treatment with dressings and antimicrobial agents does not involve donor site morbidity, which can result in pain, scarring, and long-term deviant pigmentation [13]. On the other hand, conservative treatment is associated with a longer time to re-epithelialisation and a prolonged length of hospital stay, which accounts for high costs in burn care [14, 15]. Also, it can be related to the development of hypertrophic scars and joint contractures due to delayed wound healing [14]. Additionally, early wound closure is associated with a reduced infection risk and shorter length of hospital stay, which can contribute to early rehabilitation [16, 17]. However, early excision and grafting might result in scarring of more superficially burned areas that might have been able to heal with little scarring if treated conservatively [3].

Two systematic reviews examined the evidence on the optimal treatment for deep partial-thickness burns. Ong et al. [14] included six randomized controlled trials on early versus delayed excision and grafting between 1966 and 2004. Timing of early excision ranged from less than 24 hours to 144 hours post-burn. Clinical outcomes such as mortality and blood loss were evaluated, and one of the included studies analysed cosmetics evaluated by healthcare professionals [18]. The systematic review concluded that early burn excision resulted in lower mortality and length of hospital stay, as well as increased blood transfusion requirements [14].

Miroshnychenko et al. [19] included nine randomized controlled trials comparing early burn surgery to late surgical intervention from 1982 to 2019. Early surgery was defined as excision and grafting within seven days post-burn, and delayed surgery as excision and grafting after seven days. The authors concluded that early excision and grafting may reduce length of hospital stay and mortality and might lead to better functional and cosmetic outcomes. However, the quality of evidence was low and outcomes varied between different studies [20, 21]. Again, the included studies primarily focused on clinical outcomes, and the only study that included scar quality found no statistically significant difference between both treatment groups [21].

The quality of evidence of the analysed studies in both systematic reviews is questionable. The majority of the studies date from the 1980s, which renders them outdated. Furthermore, the terms ‘excision’ and ‘excision and grafting’ seem to be used and analysed interchangeably. Therefore, this protocol describes the study that is designed to assess long-term scar quality of conservative treatment with delayed excision and grafting if needed, compared to early excision and grafting of deep partial-thickness burns in acute adult burn care. Thereby gaining more insight into the advantages and disadvantages of the treatment of these burns. In a multicentre prospective study, long-term scar quality of both treatment strategies will be analysed, and clinical and patient-reported outcomes will be examined.

## Materials and methods

### Study design

A multicentre prospective study with a 12-month follow-up period will be conducted in the three Dutch burn centres: Maasstad Hospital Rotterdam, Red Cross Hospital Beverwijk, and Martini Hospital Groningen. Patients with deep partial-thickness burns, scanned as yellow by Laser Doppler Imaging (LDI), will be monitored during 18 months; two nine-month periods with either therapy as the treatment of choice in one of the periods. This time period was chosen based on expected numbers and project logistics, such as staffing. Standard care in the Netherlands for deep partial-thickness burns is predominantly conservative treatment with delayed excision and grafting if needed. However, early excision and grafting is applied if deemed more appropriate for a specific patient. The first nine-month period (July 15^th^ 2022 to April 14^th^ 2023) this strategy is followed. In the following nine-month period (April 15^th^ 2023 to January 14^th^ 2024) clinical practice will be changed: early excision and grafting will be the primary treatment. However, in specific cases e.g. based on clinical expertise, conservative treatment can be applied. Depending on the received treatment, patients will be categorized as either the conservative treatment group with delayed excision and grafting if needed, or the early excision and grafting group.

### Protocol and registration

**Fig 1:**
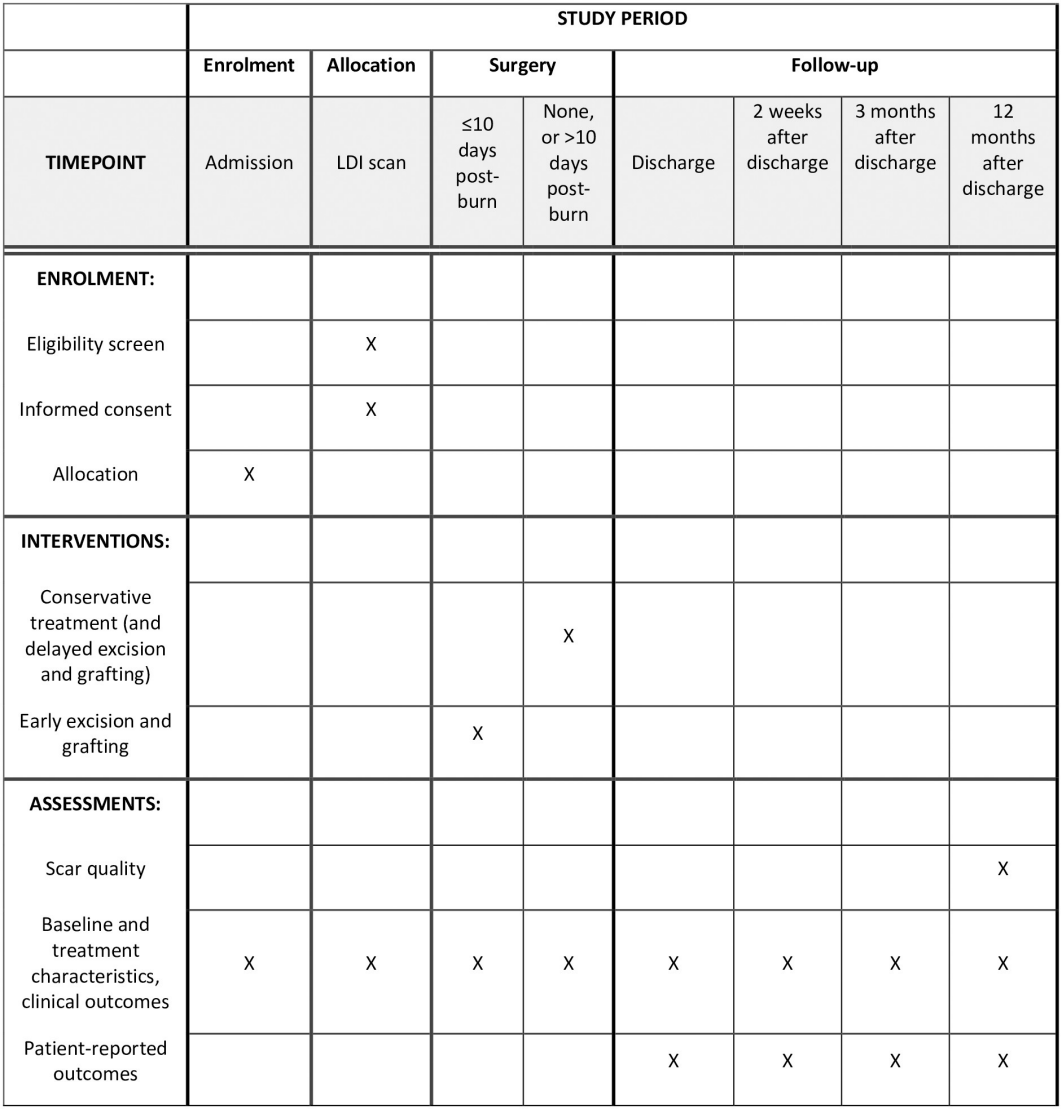
SPIRIT schedule of enrolments, interventions, and assessments.

This protocol is in accordance to the Standard Protocol Items: Recommendations for Interventional Trials (SPIRIT) guidelines [22]. The SPIRIT Figure and SPIRIT Checklist are given in Fig 1 and S1 File.

### Participants

#### Selection

Neither, randomisation nor blinding will take place. The used treatment strategy determines to which group the patient is assigned. The first group represents conservative treatment with delayed excision and grafting if needed, the second group represents early excision and grafting. If a patient is eligible for the study, a member of the research team will provide information about the study. The patient is asked for consent to take additional measurements 12 months after discharge. After reflection time and written informed consent, the patient is included.

Anyone using the protocol must consider the ethical implications of the timing of surgery in their own practice setting. To ensure patient safety, the attending physician is justified in deciding not to start or discontinue treatment from the allocated treatment, based on sound medical grounds and after consulting with the health care team. Deviation from the assigned treatment should be rare, and the reason must be thoroughly documented.

#### Criteria

Adult (≥18 years) patients with acute thermal burns who are admitted for at least 24 hours or scheduled for surgery are eligible for this study, when the criteria listed below are met.

Inclusion criteria:

- Burns with a size of >2.5 centimetres diameter;
- LDI scan indicating:
  - Deep partial-thickness burn: yellow wound area on LDI with healing potential between 14 and 21 days, or;
  - Mixed superficial and deep partial-thickness burn: mixed red and yellow wound area on LDI, with a demarcated predominantly yellow area of at least 25cm^2^, or;
  - Mixed full-thickness and deep partial-thickness burn: mixed blue and yellow wound area on LDI, with a demarcated predominantly yellow area of at least 25cm^2^.

The colour codes representing the perfusion units (PU) and healing potential are as follows [8, 23]:

- Red/pink: PU 441–2500, healing potential within 14 days;
- Yellow/green: PU 201–440, healing potential about 14 to 21 days;
- Blue: PU 0–200, healing potential more than 21 days.

Exclusion criteria:

- Frostbite injury [24];
- Burns suitable for primary closure;
- Patients unable to complete the Patient and Observer Scar Assessment Scale (POSAS) due to language restrictions or legal incompetence.

### Standard care

#### Laser Doppler Imaging

The assessment of healing potential is critical for clinical decision-making [25]. LDI is considered the most accurate method for predicting the burn wound healing potential [26]. The LDI technique is based on the Doppler principle with a laser beam that reflects moving objects, such as erythrocytes. The amount of reflection on photodiodes displays dermal perfusion on a scale, from which the healing potential is derived [27]. LDI shows an accuracy of more than 95% and is mostly used in combination with bedside clinical examination [28]. Especially for deep partial-thickness burns clinical assessment of burn depth can be challenging. Yet, an accurate burn depth assessment is important to determine the appropriate treatment.

Patients receive an LDI scan upon indication of the attending physician. The moorLDI2-Burn Imager^™^ scan (Moor Instruments, Axminster, UK) is conducted between 48 hours and five days post-burn for optimal accuracy [29]. The LDI scan procedure is standard care in the Netherlands and performed in daily clinical practice by experienced and trained personnel.

#### Burn wound area selection

Based on the healing potential, the attending physician will determine whether the patient has burns that meet the inclusion criteria. A maximum of two inclusion criteria-meeting burns per patient are followed for the study.

#### Surgical technique

Excision and grafting are carried out in accordance with the local protocol of the operating burn centre. Burns will be tangentially excised using guarded knives and/or hydrosurgery. Autografts are harvested with an electrical dermatome from a preoperatively selected donor site. Autografts will be predominantly meshed. Postoperative care is equal for both treatment groups.

### Procedures

#### Conservative treatment with delayed excision and grafting if needed

Initial conservative treatment is one of the strategies used in daily clinical practice to treat deep partial-thickness burns. Patients receive conservative treatment involving the application of dressings and antimicrobial agents. These include silver sulfadiazine (Flammazine®), cerium nitrate-silver sulfadiazine (Flammacerium®), enzymatic hydrated alginate polymers (Flaminal®), or other agents and dressings according to the local protocol of the burn centre. If wound healing is not achieved after 14 to 21 days, excision and grafting of the residual defects can be performed. This is decided based on the expert opinion of the burn specialist and in consultation with the patient.

#### Early excision and grafting

Patients initially receive abovementioned topical treatment, followed by excision and grafting of the burn in the first week to ten days post-burn.

### Primary outcome

#### Scar quality

The primary outcome measure of the study is long-term scar quality assessed with the POSAS (generic version, 3.0) 12 months after discharge. The POSAS is a widely used scar quality measurement instrument that comprises two separate scales: the Patient Scale and the Observer Scale [30]. The Patient Scale includes 17 questions about visual, tactile and sensory characteristics, and the overall opinion. The Observer Scale includes eight questions assessing the scar’s pigmentation, vascularity, surface level, surface texture, firmness, adherence, tension, and overall opinion. Each item is rated on a five-point scale. The lowest score is ‘1’, which corresponds to the situation of normal skin (i.e. normal pigmentation, no itching). Score ‘5’ equals the largest difference from normal skin (i.e. the worst imaginable scar or sensation) [30].

The Patient Scale is assessed by the patient, and two independent observers assess the Observer Scale on the study area(s). The assessment is mostly done during the standard 12-month outpatient appointment by the attending burn physician(s) and/or by members of the research team. If there is no standard 12-month outpatient appointment planned, the patient is invited to attend the clinic for the scar quality assessment, for which travel and parking expenses are reimbursed. All members of the research team and the burn physicians are trained to assess scar quality with the POSAS. Each burn centre’s highly experienced head of research provides a POSAS training on the latest version: POSAS 3.0 [30].

### Secondary outcomes

#### Clinical outcomes

Time to wound healing of study area(s): this is defined as the number of days between burn injury and re-epithelialisation of >95% of the wound surface. Progression of re-epithelialisation is assessed by an experienced burn specialist and reported after every wound inspection.

Wound infection of study area(s): the attending physician will document whether a wound infection has occurred, based on inflammation characteristics (rubor, dolor, calor, tumor, functio laesa) in combination with a positive wound culture.

As of 2009, patient and clinical characteristics are prospectively registered in the Dutch Burn Repository (DBR) R3 in the three Dutch burn centres. Clinical outcomes that will be obtained from DBR R3 are mortality (during admission), time to first excision and grafting, length of hospital stay, length of ICU stay, readmission, reoperation, blood transfusion requirements, wound complications (e.g. graft failure), sepsis, shock, reconstructive surgery, number and timing of outpatient visits. To assess the amount of clinical topical wound treatments, the length of hospital stay will be used as a proxy.

#### Patient reported outcomes

For Dutch burn care, a patient relevant value based healthcare burn core set was developed and implemented in usual care (Spronk et al, submitted). Patient reported outcomes of this value based healthcare burn core set are evaluated in the Burn centres Outcomes Registry the Netherlands (BORN) questionnaires, which are assessed at discharge (including retrospective questions), 2 weeks, 3 months, and 12 months after discharge, and annually afterwards. For all three participating burn centres, the questionnaires are implemented as usual care. The BORN questionnaires include the following topics:

- Scar quality of a scar chosen by the patient by POSAS 3.0 patient scale [30];
- Pain measured by the EuroQol 5D-5L (EQ-5D-5L) [31] pain item and the Patient-Reported Outcomes Measurement Information System (PROMIS) [32] short forms (SF) pain 1a;
- Self-care measured by the EQ-5D-5L self-care item;
- Depressive complaints assessed by the EQ-5D-5L item anxiety/depression and PROMIS SF depression 4a;
- Quality of life measured by the PROMIS Global01 and Global02;
- Post-traumatic stress symptoms measured by the Impact of Event Scale (IES-6) [33];
- Independence by a self-developed item independence;
- Physical functioning assessed by the PROMIS SF physical function 8b;
- Itching complaints measured by the itch Numeric Rating Scale (itch-NRS) [34];
- Self-management assessed by the Partners in Health (PIH) scale [35];
- Return to work or school assessed by the adjusted International Consortium for Health Outcomes Measurement (ICHOM) [36] return to work/school;

#### Patient and treatment characteristics

Demographics, burn characteristics, and treatment characteristics will be obtained from DBR R3.

Demographics: age, gender, medical history, referral, burn centre. Burn characteristics: TBSA, date of burn injury, burn localisation, burn aetiology, inhalation injury. Treatment characteristics: date of initial surgical intervention and thereby time till first surgical intervention (in days), admission on Intensive Care Unit (ICU), ventilation, topical wound treatments, and wound debridement method, type of grafting, other surgical interventions if applicable.

### Data management

Coded research data will be collected and managed in Castor Electronic Data Capture (EDC). Hard copy questionnaires and informed consent files will be maintained within locked cabinets in the burn centre, only accessible to the local research team.

### Sample size

No formal sample size calculation was performed for this prospective study. The sample size was assessed using data from DBR R3 (unpublished data, 2019) and a randomized controlled trial on LDI in patients with indeterminate depth burns in Dutch burn centres [37]. Excluding patients with superficial burns and patients with full-thickness burn injuries, and extrapolating the data to three burn centres, the expected sample size is a maximum of 204 patients for the 18-month study period.

### Statistical analysis

For the statistical analysis descriptive statistics will be used. All parameters will be tested for normality. Depending on the distribution of the data, a mean and standard deviation (normal) or a median and interquartile range (skewed) will be displayed. Differences between the two treatment strategies for the primary outcome measure scar quality and other outcomes will be analysed using two-sided T-test (normally distributed continuous data) or Mann-Whitney U-test (skewed continuous data), and two-sided Chi-square test or Fisher’s exact test (categorical data). Differences between groups in time to event outcomes (i.e. time to surgical intervention and time to wound healing) will be analysed using Kaplan-Meier regression. To correct for the possible influence of patient, burn, and burn centre characteristics on the results, multivariate linear regression (continuous data), multilevel logistic regression (categorical data), and Cox regression (time to event data) will be used. If possible, exploratory analyses will be done on the 10–14 day surgery group. Data analysis will be performed using IBM Statistical Package for the Social Sciences (SPSS) version 26 (SPSS, Chicago, IL, USA) and Python (Python Software Foundation, Delaware, USA).

### Ethical considerations and declarations

In consultation with the medical ethics committee, it was agreed that the treatment strategies are part of usual care. Therefore, a medical ethics request was made solely for the conduction of the POSAS 12 months after discharge. The Medical research Ethics Committees United (MEC-U) has confirmed that the Medical Research Involving Human Subjects Act (WMO) does not apply to this study (W23.039) on March 14^th^ 2023. The study protocol was approved by the local review boards of all participating hospitals: Maasstad Hospital Rotterdam (L2023027), Red Cross Hospital Beverwijk (23.010), and Martini Hospital Groningen (2023–080).

### Study status and timeline

Data collection from DBR R3 and BORN started on July 15^th^ 2022. Inclusion of participants started after approval of the local review boards of all participating hospitals on June 25^th^ 2023. The first patient inclusion and 12-month POSAS was conducted on June 30^th^ 2023. Data collection and participant selection is expected to be completed in January 2025.

## Discussion

In this protocol we described the aim and specific design attributes of our study to address the gaps in understanding regarding the timing of surgical intervention after deep partial-thickness burn injury. In particular, we intend to compare long-term scar quality, clinical outcomes, and patient-reported outcomes evident after conservative treatment with delayed excision and grafting if needed, and early excision and grafting of deep partial-thickness burns in Dutch burn care. While patient-reported outcomes on this topic are scarce, this study focuses on long-term patient-reported outcomes that will be measured using the POSAS 3.0 and the BORN [38]. POSAS is a unique scar quality assessment tool, as it not only includes the professional’s perspective but also incorporates the patient’s view [39]. BORN provides a wide range of outcomes that were considered important by patients, healthcare professionals, and researchers (Spronk et al, submitted). This will provide insight into the outcomes related to timing of surgical intervention to enable healthcare providers to offer patients more in-depth information when surgery is discussed. The study findings are aimed to support shared decision making in person-centred burn care.

A strength of this study is the multicentre design which improves generalizability and applicability to similarly resourced burn care facilities. Additionally, the homogeneity of the included study burn wound areas is another strength of the study. The followed burns are all deep partial-thickness burns with a comparable healing potential.

A possible limitation might be the absence of donor site quality as an outcome. However, it was deliberately chosen not to include this, since ample literature regarding this topic is available and a recent study on long-term patient-reported scar quality of donor sites was conducted in one of the participating burn centres [13].

Regarding considerations for collaborative research: by publishing this study protocol, we aim to encourage authors from other countries to conduct similar studies. Multisite studies can enhance the generalisability of findings and improve evidence-based burn care [40]. This study protocol leaves room for translation to a randomized controlled trial (RCT). However, we decided not to conduct an RCT due to the niche sector of burn care and to increase the external validity of our results by collecting real-world data. Therefore, we encourage other authors to use real-world data for answering the optimal surgical timing research question. The POSAS was chosen for the direct assessment of scar quality because of its reliability, validity, and feasibility. It is recommended to use the POSAS for long-term scar quality assessment, provided that personnel are trained in conducting it. One could choose for standardized images with a ruler as outcome measure, but this was not selected in this study due to their difficult comparability and the availability of trained POSAS personnel.

The findings of the described study add to the ongoing worldwide debate on the optimal treatment strategy for deep partial-thickness burns. Furthermore, the results of this study will be included in a cost-effectiveness study and in the development of a decision aid for shared decision making on timing of surgery in acute burns. The decision aid will support the patient and healthcare provider in the decision for the optimal treatment based on the patients’ goals and preferences.

## Supporting information

S1 File SPIRIT checklist. (DOC)

S2 File Study protocol. (DOCX)

## References

[1] Greenhalgh DG. Management of Burns. N Engl J Med. 2019;380(24):2349–59. doi: 10.1056/NEJMra1807442 31189038

[2] Hebron C, Mehta K, Stewart B, Price P, Potokar T. Implementation of the World Health Organization Global Burn Registry: Lessons Learned. Ann Glob Health. 2022;88(1):34. doi: 10.5334/aogh.3669 35646613 PMC9122007

[3] Goei H, van der Vlies CH, Hop MJ, Tuinebreijer WE, Nieuwenhuis MK, Middelkoop E, et al. Long-term scar quality in burns with three distinct healing potentials: A multicenter prospective cohort study. Wound Repair Regen. 2016;24(4):721–30. doi: 10.1111/wrr.12438 27102976

[4] Jeschke MG, van Baar ME, Choudhry MA, Chung KK, Gibran NS, Logsetty S. Burn injury. Nat Rev Dis Primers. 2020;6(1):11. doi: 10.1038/s41572-020-0145-5 32054846 PMC7224101

[5] Karim AS, Shaum K, Gibson ALF. Indeterminate-Depth Burn Injury-Exploring the Uncertainty. J Surg Res. 2020;245:183–97. doi: 10.1016/j.jss.2019.07.063 31421361 PMC8711117

[6] Jaspers MEH, van Haasterecht L, van Zuijlen PPM, Mokkink LB. A systematic review on the quality of measurement techniques for the assessment of burn wound depth or healing potential. Burns. 2019;45(2):261–81. doi: 10.1016/j.burns.2018.05.015 29941159

[7] Buz A, Gorgulu T, Olgun A, Kargi E. Efficacy of glutathione mesotherapy in burns: an experimental study. Eur J Trauma Emerg Surg. 2016;42(6):775–83. doi: 10.1007/s00068-015-0607-8 26614529

[8] Jaspers MEH. Beyond the skin: new insights in burn care 2018.

[9] Committee IPG, Steering S, Advisory S. ISBI Practice Guidelines for Burn Care. Burns. 2016;42(5):953–1021. doi: 10.1016/j.burns.2016.05.013 27542292

[10] Singer AJ, Toussaint J, Chung WT, McClain SA, Raut V, Rosenberg L. Early versus Delayed Excision and Grafting of Full-Thickness Burns in a Porcine Model: A Randomized Study. Plast Reconstr Surg. 2016;137(6):972e–9e. doi: 10.1097/PRS.0000000000002161 27219266

[11] Yoshino Y, Ohtsuka M, Kawaguchi M, Sakai K, Hashimoto A, Hayashi M, et al. The wound/burn guidelines—6: Guidelines for the management of burns. J Dermatol. 2016;43(9):989–1010. doi: 10.1111/1346-8138.13288 26971391

[12] Hanna K, Ditillo M, Joseph B. The role of frailty and prehabilitation in surgery. Curr Opin Crit Care. 2019;25(6):717–22. doi: 10.1097/MCC.0000000000000669 31689246

[13] Legemate CM, Ooms PJ, Trommel N, Middelkoop E, van Baar ME, Goei H, et al. Patient-reported scar quality of donor-sites following split-skin grafting in burn patients: Long-term results of a prospective cohort study. Burns. 2021;47(2):315–21. doi: 10.1016/j.burns.2020.12.005 33419665

[14] Ong YS, Samuel M, Song C. Meta-analysis of early excision of burns. Burns. 2006;32(2):145–50. 16414197 10.1016/j.burns.2005.09.005

[15] Hop MJ, Polinder S, Middelkoop E, van BM. Costs of Burn Care: A Systematic Review. Value Health. 2014;17(7):A606. 10.1016/j.jval.2014.08.2111 27202101

[16] Wong L, Rajandram R, Allorto N. Systematic review of excision and grafting in burns: Comparing outcomes of early and late surgery in low and high-income countries. Burns. 2021;47(8):1705–13. doi: 10.1016/j.burns.2021.07.001 34303572

[17] Singer AJ, Boyce ST. Burn Wound Healing and Tissue Engineering. J Burn Care Res. 2017;38(3):e605–e13. 28328668 10.1097/BCR.0000000000000538 PMC5461657

[18] Engrav LH, Heimbach DM, Reus JL, Harnar TJ, Marvin JA. Early excision and grafting vs. nonoperative treatment of burns of indeterminant depth: a randomized prospective study. J Trauma. 1983;23(11):1001–4. doi: 10.1097/00005373-198311000-00007 6355500

[19] Anna Miroshnychenko KK, Bram Rochwerg, Sophocles Voineskos. Comparison of early surgical intervention to delayed surgical intervention for treatment of thermal burns in adults: A systematic review and meta-analysis. Burns. 2021;5(2):67–77.

[20] Herndon DN, Barrow RE, Rutan RL, Rutan TC, Desai MH, Abston S. A comparison of conservative versus early excision. Therapies in severely burned patients. Ann Surg. 1989;209(5):547–52; discussion 52–3. doi: 10.1097/00000658-198905000-00006 2650643 PMC1494069

[21] Mohammadi AA, Bakhshaeekia AR, Marzban S, Abbasi S, Ashraf AR, Mohammadi MK, et al. Early excision and skin grafting versus delayed skin grafting in deep hand burns (a randomised clinical controlled trial). Burns. 2011;37(1):36–41. doi: 10.1016/j.burns.2010.02.005 20537468

[22] Chan AW, Tetzlaff JM, Altman DG, Laupacis A, Gotzsche PC, Krleza-Jeric K, et al. SPIRIT 2013 statement: defining standard protocol items for clinical trials. Ann Intern Med. 2013;158(3):200–7. doi: 10.7326/0003-4819-158-3-201302050-00583 23295957 PMC5114123

[23] Monstrey SM, Hoeksema H, Baker RD, Jeng J, Spence RS, Wilson D, et al. Burn wound healing time assessed by laser Doppler imaging. Part 2: validation of a dedicated colour code for image interpretation. Burns. 2011;37(2):249–56. 21084164 10.1016/j.burns.2010.08.013

[24] Moor Instruments A, UK. MoorLDI2-BI Training Manual Burns Software. 4 ed2022.

[25] Heyneman A, Hoeksema H, Vandekerckhove D, Pirayesh A, Monstrey S. The role of silver sulphadiazine in the conservative treatment of partial thickness burn wounds: A systematic review. Burns. 2016;42(7):1377–86. doi: 10.1016/j.burns.2016.03.029 27126813

[26] Claes KEY, Hoeksema H, Robbens C, Verbelen J, Dhooghe N, De Decker I, et al. The LDI Enigma Part II: Indeterminate depth burns, man or machine? Burns. 2021;47(8):1773–82. doi: 10.1016/j.burns.2021.01.015 34696950

[27] Hop MJ, Hiddingh J, Stekelenburg C, Kuipers HC, Middelkoop E, Nieuwenhuis MK, et al. Cost-effectiveness of laser Doppler imaging in burn care in the Netherlands. BMC Surg. 2013;13:2. 23369360 10.1186/1471-2482-13-2 PMC3574826

[28] Claes KEY, Hoeksema H, Robbens C, Verbelen J, Dhooghe NS, De Decker I, et al. The LDI Enigma, Part I: So much proof, so little use. Burns. 2021;47(8):1783–92. doi: 10.1016/j.burns.2021.01.014 33658147

[29] Hoeksema H, Van de Sijpe K, Tondu T, Hamdi M, Van Landuyt K, Blondeel P, et al. Accuracy of early burn depth assessment by laser Doppler imaging on different days post burn. Burns. 2009;35(1):36–45. 18952377 10.1016/j.burns.2008.08.011

[30] Carriere ME, Mokkink LB, Tyack Z, Westerman MJ, Pijpe A, Pleat J, et al. Development of the Patient Scale of the Patient and Observer Scar Assessment Scale (POSAS) 3.0: a qualitative study. Qual Life Res. 2023;32(2):583–92. 36355319 10.1007/s11136-022-03244-6 PMC9911488

[31] Herdman M, Gudex C, Lloyd A, Janssen M, Kind P, Parkin D, et al. Development and preliminary testing of the new five-level version of EQ-5D (EQ-5D-5L). Qual Life Res. 2011;20(10):1727–36. 21479777 10.1007/s11136-011-9903-x PMC3220807

[32] Cella D, Yount S, Rothrock N, Gershon R, Cook K, Reeve B, et al. The Patient-Reported Outcomes Measurement Information System (PROMIS): progress of an NIH Roadmap cooperative group during its first two years. Med Care. 2007;45(5 Suppl 1):S3–S11. 10.1097/01.mlr.0000258615.42478.55 PMC2829758 17443116

[33] Hosey MM, Leoutsakos JS, Li X, Dinglas VD, Bienvenu OJ, Parker AM, et al. Screening for posttraumatic stress disorder in ARDS survivors: validation of the Impact of Event Scale-6 (IES-6). Crit Care. 2019;23(1):276. 31391069 10.1186/s13054-019-2553-z PMC6686474

[34] Kimel M, Zeidler C, Kwon P, Revicki D, Stander S. Validation of Psychometric Properties of the Itch Numeric Rating Scale for Pruritus Associated With Prurigo Nodularis: A Secondary Analysis of a Randomized Clinical Trial. JAMA Dermatol. 2020;156(12):1354–8. 32936233 10.1001/jamadermatol.2020.3071 PMC7495327

[35] W Battersby M, Ask A., Reece M. M, Markwick M. J, Collins J P. The Partners in Health scale: The development and psychometric properties of a generic assessment scale for chronic condition self-management. Australian Journal of Primary Health. 2003;9(3):41.

[36] Terwee CB, Zuidgeest M, Vonkeman HE, Cella D, Haverman L, Roorda LD. Common patient-reported outcomes across ICHOM Standard Sets: the potential contribution of PROMIS(R). BMC Med Inform Decis Mak. 2021;21(1):259. 34488730 10.1186/s12911-021-01624-5 PMC8420145

[37] Hop MJ, Stekelenburg CM, Hiddingh J, Kuipers HC, Middelkoop E, Nieuwenhuis MK, et al. Cost-Effectiveness of Laser Doppler Imaging in Burn Care in The Netherlands: A Randomized Controlled Trial. Plast Reconstr Surg. 2016;137(1):166e–76e. 10.1097/PRS.0000000000001900 26710049

[38] Venkatesh K, Henschke A, Lee RP, Delaney A. Patient-centred outcomes are under-reported in the critical care burns literature: a systematic review. Trials. 2022;23(1):199. 35246209 10.1186/s13063-022-06104-3 PMC8896280

[39] Draaijers LJ, Tempelman FR, Botman YA, Tuinebreijer WE, Middelkoop E, Kreis RW, et al. The patient and observer scar assessment scale: a reliable and feasible tool for scar evaluation. Plast Reconstr Surg. 2004;113(7):1960–5; discussion 6–7. 15253184 10.1097/01.prs.0000122207.28773.56

[40] Cuttle L, Fear M, Wood FM, Kimble RM, Holland AJA. Management of non-severe burn wounds in children and adolescents: optimising outcomes through all stages of the patient journey. Lancet Child Adolesc Health. 2022;6(4):269–78. 35051408 10.1016/S2352-4642(21)00350-3

