# Correction: Timing of surgery in acute deep partial-thickness burns: A study protocol

**DOI:** 10.1371/journal.pone.0328380

**Published:** 2025-07-15

**Authors:** Roos F. C. Salemans, Denise van Uden, Margriet E. van Baar, Tjitske M. Haanstra, Carine H. M. van Schie, Paul P. M. van Zuijlen, Ymke Lucas, Sonja M. H. J. Scholten-Jaegers, Annebeth Meij-de Vries, Fiona M. Wood, Dale W. Edgar, Inge Spronk, Cornelis H. van der Vlies, National Burn Care, Education & Research group, the Netherlands

There are errors in the author affiliations. The correct affiliations are as follows:

Roos F.C. Salemans^1,2^, Denise van Uden^2^, Margriet E. van Baar^3,4^, Tjitske M. Haanstra^5,6^, Carine H. M. van Schie^6^, Paul P. M. van Zuijlen^7,8,9,10^, Ymke Lucas^2^, Sonja M. H. J. Scholten-Jaegers^11^, Annebeth Meij-de Vries^7,10,12^, Fiona M. Wood^13,14,^ Dale W. Edgar^13,14,15,16,17^, Inge Spronk^3,4,6^, Cornelis H. van der Vlies^1,18^, National Burn Care, Education & Research group, the Netherlands

**1** Trauma Research Unit Department of Surgery, Erasmus MC, University Medical Centre Rotterdam, Rotterdam, the Netherlands, **2** Burn Centre, Maasstad Hospital, Rotterdam, the Netherlands, **3** Association of Dutch Burn Centres, Maasstad Hospital, Rotterdam, the Netherlands, **4** Erasmus MC, University Medical Centre Rotterdam, Department of Public Health, Rotterdam, the Netherlands, **5** Department of Dermal Therapy, Faculty of Health, Nutrition & Sport, The Hague University of Applied Sciences, The Hague, the Netherlands; Research Group Relational Care, Centre of Expertise Health Innovation, The Hague University of Applied Sciences, The Hague, the Netherlands, **6** Dutch Burns Foundation, Beverwijk, the Netherlands, **7** Burn Centre, Red Cross Hospital, Beverwijk, the Netherlands, **8** Department of Plastic and Reconstructive Surgery, Red Cross Hospital, Beverwijk, the Netherlands, **9** Department of Plastic Reconstructive and Hand Surgery, Amsterdam Movement Sciences Institute, Amsterdam UMC, location VUmc, the Netherlands, **10** Paediatric Surgical Centre, Emma’s Children’s Hospital, Amsterdam UMC, location AMC, the Netherlands, **11** Burn Centre, Martini Hospital, Groningen, the Netherlands, **12** Department of Surgery, Red Cross Hospital, Beverwijk, the Netherlands, **13** Fiona Wood Foundation, Fiona Stanley Hospital, Murdoch, Western Australia, Australia, **14** State Adult Burn Unit, Fiona Stanley Hospital, South Metropolitan Health Service, Murdoch, Western Australia, Australia, **15** Institute for Health Research, Burn Injury Research Node, The University of Notre Dame Australia, Fremantle, Western Australia, Australia, **16** Burn Injury Research Unit, Faculty of Medicine and Dentistry, University of Western Australia, Crawley, Western Australia, Australia, **17** Safety and Quality Unit, Armadale Kalamunda Group Health Service, East Metropolitan Health Service, Mt Nasura, Western Australia, Australia, **18** Department of Trauma and Burn Surgery, Maasstad Hospital, Rotterdam, the Netherlands.
