# Supplementary material for: Timing of surgery in acute deep partial-thickness burns: A study protocol
Source: PLoS One. 2024 Mar 11;19(3):e0299809. doi: 10.1371/journal.pone.0299809 (PMC10927102; doi:10.1371/journal.pone.0299809)
Supplement: S2 File — (DOCX) [file pone.0299809.s002.docx]

**Onderzoeksprotocol niet WMO-plichtig onderzoek Rotterdam**

| STUDY DETAILS | | | | |
| --- | --- | --- | --- | --- |
| Study title | Outcomes on timing of surgery in deep partial-thickness burns | | | |
| Acronym / short title | TOS (timing of surgery) study | | | |
| Date / Version | 10-03-2023  Version 2.1 | | | |
| Study design | ☐ Retrospective study  X (Partial) prospective study  ☐ Other: | | | |
| Provider/sponsor | Consortium Samenwerkende Brandwondencentra Nederland, location  Maasstad Hospital Rotterdam | | | |
| Research team | Name | | | Role |
|  | 1 | A/prof. C.H. van der Vlies | | Principal Investigator |
|  | 2 | R.F.C. Salemans | | Coordinating investigator |
|  | 3 | M.E. van Baar | | Researcher |
|  | 4 | D. van Uden | | Researcher |
|  | 5 | S.M. Thambiturai | | Researcher |
|  | 6 | M.C. Heijblom | | Research nurse |
|  | 7 | H.W.C. Hofland | | Nurse scientist |
|  | 8 | M.A.C.M de Craen | | Research nurse |
| Submitter | Name: Roos Salemans  Tel: 06 28 29 77 73  E-mail: SalemansR@maasstadziekenhuis.nl | | | |
| (Local) principal investigator | Name: Kees van der Vlies  Tel: (010) 291 34 28  E-mail: VliesC@maasstadziekenhuis.nl | | | |
| The study is being conducted as part of: | ☐ | | General scientific research | |
|  | X | | PhD research | |
|  | ☐ | | Scientific internship/bachelor’s or master’s thesis | |
|  | ☐ | | Other: | |

# Table of contents

SAMENVATTING ................................................................................................................................... 4

1. INTRODUCTIE ................................................................................................................................ 4
2. ONDERZOEKSVRAAG/ ONDERZOEKSDOEL .................................................................................... 5
3. METHODEN ................................................................................................................................... 5
   1. Mono- of multicenter studie ................................................................................................. 5
   2. Studiedesign.......................................................................................................................... 5
   3. Procedure en interventie (indien van toepassing) ................................................................. 6
   4. Duur van de studie ................................................................................................................ 6
   5. Werving en selectie van proefpersonen ................................................................................ 6
   6. Dataverzameling: variabelen en meetmethoden .................................................................. 7
   7. Data-analyse ......................................................................................................................... 8
4. ETHISCHE OVERWEGINGEN .......................................................................................................... 9
   1. Niet WMO verklaring ............................................................................................................ 9
   2. Belasting en vergoeding voor de proefpersoon .................................................................... 9
   3. Toestemming proefpersoon .................................................................................................. 9
5. DATAMANAGEMENT & PRIVACY................................................................................................. 10
   1. Dataopslag, beveiliging en toegang tijdens onderzoek ....................................................... 10
   2. Dataverwerking ................................................................................................................... 12
   3. Data delen ........................................................................................................................... 13
   4. Hoe lang worden de data bewaard? ................................................................................... 14
6. VALORISATIE EN PUBLICATIE....................................................................................................... 14
   1. Valorisatie ........................................................................................................................... 14
   2. Publicatie ............................................................................................................................ 14
7. REFERENTIES ............................................................................................................................... 14

| SAMENVATTING (DUTCH) |
| --- |
| De optimale behandeling van gedeeltelijk diepe brandwonden blijft een onderwerp van discussie in de brandwondenzorg. Op dit moment zijn er twee behandelstrategieën die worden toegepast bij deze brandwonden. Namelijk 1) conservatief beleid met een huidtransplantatie van de restdefecten na 14 tot 21 dagen, indien nodig of 2) een huidtransplantatie binnen 7 dagen na het brandwondongeval. Deze laatste behandeling wordt ook wel vroeg opereren genoemd. Beide behandelstrategieën hebben voor- en nadelen, maar deze zijn nog niet volledig bekend en er is tot nu toe nog weinig aandacht geweest voor patiënt-relevante uitkomsten.  Om de voor- en nadelen en patiënt-relevante uitkomsten van beide behandelstrategieën beter in  kaart te brengen, wordt een cluster cross-over studie in de drie brandwondencentra van Nederland uitgevoerd. Gedurende 9 maanden wordt de huidige zorg, het overwegend conservatieve beleid, voortgezet voor patiënten met gedeeltelijk diepe brandwonden. In de daaropvolgende 9 maanden zal vroeg opereren de standaardbehandeling worden. Data worden ontleend aan de klinische Nederlandse Brandwonden Registratie (NBR) R3 en uitkomstenregistratie Burn centres Outcomes Registry the Netherlands (BORN). Daarnaast worden littekens na 12 maanden na ontslag beoordeeld met de Patient and Observer Scar Assessment Scale (POSAS) 3.0.  De opgedane inzichten zullen bijdragen aan geoptimaliseerde acute brandwondenzorg en zullen uiteindelijk gebruikt worden om een keuzehulp te ontwikkelen waarin informatie op maat wordt aangeboden. Hiervoor wordt een aparte aanvraag ingediend. |
| INTRODUCTION |
| Burn injuries are a major health concern worldwide, causing death and disability. Globally the incidence of burn injuries is nearly nine million annually, and approximately one hundred twenty thousand people die of burn-related injuries every year [1]. In 2018 one thousand fifty-five patients were admitted with burns in the Netherlands, of which almost half were admitted or transferred to a burn center [2].    There are different types of burn wounds, based on their depth and inherently their healing time. With full thickness burns the dermis and epidermis are damaged and early excision and grafting is indicated. Superficial partial thickness burns mostly heal with conservative wound care and no surgery is needed. Deep partial-thickness burns extend into the dermis and healing time is estimated to be two to three weeks [3]. With these burns however, no consensus has been reached on the best treatment strategy.    For deep partial-thickness burns, there are two main treatment strategies, and both are currently applied in Dutch burn care. One of the two main treatment strategies is conservative therapy with dressings and topical antimicrobial and antiseptic agents, after which spontaneous healing is awaited. If spontaneous healing is not reached after two to three weeks, delayed skin grafting is applied to obtain wound closure. The other treatment strategy is early excision and skin grafting, where surgery and skin grafting is performed within seven days after the burn injury.  These strategies have pros and cons with little known on patient-relevant outcomes. Assumed advantages of conservative treatment are preserving healthy tissue and no donor site morbidity. Disadvantages of conservative treatment may include painful wound care procedures for weeks, prolonged hospital stay, and delayed rehabilitation. Disadvantages of early skin grafting may include possible overly invasive grafting, blood loss and morbidity of skin grafts, whereas advantages might include less (painful) wound dressing changes, reduced infection risk, shorter hospital stay, and earlier rehabilitation.    Currently, no high-quality evidence is available on which treatment strategy is superior. A systematic review and meta-analysis stated that early excision and grafting may reduce length of hospital stay and mortality and leads to better functional and cosmetic outcomes [4]. However, the quality of evidence is low, and outcomes vary between different studies, as some demonstrate no difference in mortality or functional outcome [5, 6].  Most studies concentrate on clinical outcomes like mortality, length of hospital stay, transfusion requirements, functional outcome, and graft take [4, 7]. However, the important outcome scar quality is rarely assessed. In the few studies evaluated, no statistically significant difference between both treatment groups were found for scar quality [4, 6].  One article included patient-reported outcomes such as average time off work and return to work.  However, this is a study from 1983, so results may be outdated [8].  Overall, there is a lack of knowledge on patient-relevant outcomes for both treatment strategies, including scar quality. Therefore, we aim to assess clinical and patient-relevant outcomes after early skin grafting versus conservative treatment. |

| RESEARCH QUESTION/ RESEARCH AIM |
| --- |
| The aim of this project is to optimize person-cent red acute burn care by analyzing the clinical and patient-relevant outcomes and scar quality of deep partial-thickness burns after early excision and grafting versus conservative treatment. |
| METHODS |
| Mono- or multicenter study |
| ☐ Monocenter study  X Multicenter study    Participating centers and local principal investigator:   1. Burn center Maasstad Hospital Rotterdam: A/prof C.H. van der Vlies 2. Burn center Red Cross Hospital Beverwijk: Dr. Ir. A. Pijpe 3. Burn center Martini Hospital Groningen: Dr. M.K. Nieuwenhuis |
| Study design |
| Clinical and patient relevant outcomes of both clusters will be extracted from Dutch Burn  Repository (DBR) R3 and the Burn centers Outcomes Registry Netherlands (BORN). Data in DBR R3 are collected during admission and follow up. Data from BORN are collected at discharge, 2 weeks after discharge, and 3 and 12 months after discharge. This is all part of standard care and already implemented in Dutch burn care. Data of the cluster cross-over study will be completed with the assessment of scar quality with the Patient and Observer Scar Assessment Scale (POSAS) 3.0, in a single measurement moment at one-year post discharge. Quantitative data analysis will be used to compare (above mentioned) data of patients with deep partial-thickness burn wounds who underwent delayed skin grafting to patients with deep-partial thickness burn wounds who underwent early skin grafting. |

| Procedure and intervention (if applicable) |
| --- |
| In the cluster cross-over study, no intervention will take place. In our current care, both treatment strategies are applied, with conservative treatment being carried out most often. The decision for a strategy is mostly based on the clinician’s preference. Patients in cluster 1 will predominantly receive conventional treatment of deep partial-thickness burns by applying dressings and antimicrobial topical agents with excision and grafting of residual defects after 14-21 days, if needed. After 9 months, the second treatment strategy, early skin grafting within 7 days post burn, will predominantly be applied to the subsequent cluster (cluster 2).    Patient-relevant outcomes on treatment strategies will be derived from BORN and R3, a value based healthcare (VHBC) burn core set, which is collected as part of standard care. The measurement of scar quality of specific wounds by patients and observers (POSAS 3.0) is not part of our standard care. Patients with deep partial-thickness burn wounds, will be asked to participate in the study by the treating healthcare professional, after which informed consent will be requested by a member of the research team or treating healthcare professional. The POSAS  3.0 consists of two scales: the Observer Scale and the Patient Scale filled in by the patient. The Observer Scale will be conducted twice: by an experienced and independent healthcare professionals and researcher. This will be done during a regular outpatient follow-up visit one-year post discharge and will take the patient about 10 minutes extra time. |
| Duration of the study |
| Start date: 15-07-2022    The 15^th^ of July 2022 we started collecting data using the updated Dutch Burn Repository (DBR) R3 and the Burn centers Outcomes Registry Netherlands (BORN). In these registries patient-relevant outcomes on treatment strategies in standard care are available. The expected date of the first regular outpatient follow-up visit one-year post discharge is 15-07-2023.    Expected end date: 15-01-2025 |
| Recruitment and selection of participants |
| Screening/selection |
| BORN is offered to all burn patients who are admitted for more than 24 hours, underwent surgery, or might benefit from participating in the PROMS system. Patients are included in the NBR R3 registry in case of treatment in a Dutch burn center. For the selection of patients with deep partial thickness burn wounds the Laser Doppler Imaging (LDI) is used, requested and reviewed by the attending physician. When the LDI indicates a wound that meets the criteria (deep-partial thickness burn), the patient is eligible for the study. The treating healthcare professional obtains permission for the approach of a member of the research team to inform the patient about participation in the study and subsequently will start the informed consent procedure. |

| Study population | | | | | |
| --- | --- | --- | --- | --- | --- |
| The study population consists of adult patients with acute deep partial-thickness burns. The burn population is very diverse. Patients of all ages, sexes, cultural backgrounds, and socioeconomic status are admitted to the burn centers. | | | | | |
| Inclusion criteria | | | | | |
| - Patient with acute burn wounds, aged ≥18 years - Admitted to the burn center for >24 hours or planned for burn surgery - Burn wound with a size > €2 coin - LDI scan indicating:   - Deep partial-thickness burns (yellow wound, healing potential between 14 and 21 days)   - Mixed deep partial-thickness and full-thickness burns (mixed yellow and blue wound areas), with a defined yellow area of at least 25cm^2^   - Mixed superficial and deep partial-thickness burns (mixed red and yellow wound areas), with a defined yellow area of at least 25cm^2^ | | | | | |
| Exclusion criteria | | | | | |
| - Frostbite injuries, electrical burns, chemical burns - Burn wounds suitable for primary closure | | | | | |
| Sample size | | | | | |
| The sample size of the cluster cross-over study is based on patient numbers of 2019 and data from a study with a similar population at Maasstad Burn Centre Rotterdam. Excluding  patients with superficial burns and patients with full-thickness burn injuries, and extrapolating the data to three burn centers the expected sample size is 102 patients per cluster. Therefore, we expect to include approximately 204 patients in total.    No formal sample size calculation is performed. | | | | | |
| Data collection: variables and measurement methods | | | | | |
| Primary outcome | | | | | |
| The primary outcome of this study is the patient and observer reported scar quality one-year post discharge determined by the POSAS 3.0. | | | | | |
| Variables and measurements overview | | | | | |
|  | **Variable** | **Instrument** | **Outcome measures** | **Measure moment** |  |
|  | Patient characteristics | DBR R3 | Age, sex, burn center, referral, medical history | During admission |  |
|  | Burn injury characteristics | DBR R3 | Date of burn injury, Total Body Surface Area (TBSA), date initial surgery, inhalation injury, burn wound localization and etiology, intensive care | During admission |  |

|  |  | |  | unit (ICU) admission, ventilation |  |  |
| --- | --- | --- | --- | --- | --- | --- |
|  | Treatment characteristics | | DBR R3 | Wound debridement method, transplantation method, other surgical  interventions (e.g. amputation), topical wound treatment | During admission and follow-up |  |
|  | Clinical  outcomes | | DBR R3 | Mortality (during admission), readmission, reoperation, blood transfusion requirements, time to wound healing, complications wound (e.g. wound infection, graft failure), sepsis, plastic/reconstructive surgery, length of hospital stay, length of ICU  stay, number and timing of outpatient visits | During admission and follow-up |  |
|  | Reported scar quality | | Patient and  Observer Scar  Assessment Scale  (POSAS) 3.0 | Scar quality from patient and clinician (observer) view | 1-year after discharge |  |
|  | Patient reported outcomes | | BORN version 3.0 or 3.1: Patient Reported Outcome  Measurement  Information System  (PROMIS), EuroQol  5D (EQ-5D) | Physical health (PROMs), shared decision making (control preference scale), posttraumatic stress disorder (PTSD) complaints (impact of event scale-6), autonomy (EuroQol 5D), physical functioning (PROMs SF physical functioning), pain (PROMs SF pain), pruritis (itch-NRS), return to work/school, depressive complaints  (PROMS SF depression) | At discharge, 1-2 weeks after discharge, 3- and 12months postburn or after discharge |  |
| Standardization | | | | | | |
| Not applicable. | | | | | | |
| Data analysis | | | | | | |
| Data inspection | | | | | | |
| Data will be checked on quality and completeness, and in the statistical analysis extreme data will be taken into account (e.g. non-normal distribution analysis, non-parametric inferential statistical methods, log transformation method). | | | | | | |
| Analyses | | | | | | |
| First, descriptive statistics will be used. All parameters will be tested whether they are normally distributed or not. Depending on the outcome, a mean and standard deviation or a median and interquartile range will be displayed. Differences in scar quality 12 months post discharge between treatment strategies will be analyzed using univariate linear regression, with a correction for burn center. Differences in time to wound healing, surgical decision, and surgery between groups will be analyzed with mixed model analysis. Three level models will be used for burn center, patients, and wound characteristics.. The possible influence of patient and burn characteristics on the results will be analyzed by multivariate analysis techniques.  The length of hospital stay will be used as a proxy for the amount of clinical topical wound treatments. | | | | | | |
| Software program | | | | | | |
| Data will be collected in Castor and Excel, and statistical analysis will be done in SPSS and R. | | | | | | |
| ETHICAL CONSIDERATIONS | | | | | | |
| Non WMO declaration | | | | | | |
| A non WMO declaration will be requested at the Medical Ethics Review Committee (METC) MECU. | | | | | | |
| Burden and compensation for the participant | | | | | | |
| Information from the registration of patient and wound characteristics and progress, as well as standardized questionnaires, will be used for this study. Since this is part of standard care, this will not be an additional burden for the patient. However, the scar quality assessment one-year after discharge in part 1 is additional for this study. Completion of the POSAS questionnaire takes approximately 10-15 minutes, for both the patient and the observer part and will be done during a regular clinical follow-up visit. | | | | | | |
| Informed consent | | | | | | |
| Is informed consent requested? | | X Yes (Vul optie A ‘Informed Consent Procedure’ in)  ☐ No (Vul optie B ‘Toestemming wordt niet gevraagd’) | | | | |
| Option A: Informed Consent procedure | | | | | | |
| Participant approach | | The informed consent procedure is set up for the additional data collection on scar quality 12 months post discharge. All other data will be derived from the standard care registrations for which no informed consent is needed. The treating healthcare professional (physician or aftercare nurse) asks the patient if he/she is interested in participating in this study. If the patient gives consent for approach, a member of the research team will provide the patient with oral and written information about the study and an informed consent form will be signed. This includes consent to obtain patient characteristics. In addition, an English version of the informed consent will be available. | | | | |
| Informing participant | | A patient will be informed about the content of the study orally and by the participant information form (PIF). The form will be available in Dutch and English with an explanatory note for low literates. There is opportunity for the patient to ask members of the research team questions about the study. When questions arise during follow-up, members of the research team can be reached by phone or email. Contact information is provided in the PIF. | | | | |

| Reflection period | A patient will be asked for participation in the period between the completion of the LDI scan and one-year follow-up. Allowing the patient ample time for reflection before participation. |
| --- | --- |
| Signing informed consent | In the period between completion of the LDI scan and one-year follow-up, the participating patient will be asked to sign the informed consent form. Depending on burn center preferences and logistical capabilities, this will be done during hospital stay or during an outpatient clinic appointment. The form will also be completed and signed by a member of the research team. |
| DATA MANAGEMENT & PRIVACY | |
| Data storage, security, and access during study | |
| Hard copy research data | |
| Where will hard copy research data (e.g. paper questionnaires and IC forms) be stored?  Hard copy questionnaires and consent files will be kept in folders located in a cabinet at the burn center. The cabinet will be locked and only members of the research team have access. | |
| Which persons have access to this storage area?  This should be at least two people because of access to the data in case of absence, illness, leaving the institution, etc.  All members of the local research team have access to the local files.  In the burn center Maasstad Hospital Rotterdam this includes:  C.H. van der Vlies (principal investigator), R.F.C. Salemans (coordinating investigator), M.E. van Baar (researcher), D. van Uden (researcher), S.M. Thambithurai (researcher), M.C Heijblom (research nurse), H.W.C. Hofland (nurse scientist), M.A.C.M de Craen (research nurse). | |
| How is it ensured that no one other than the authorized persons mentioned under 5.1.2 has access to the hard copy research data?    The key of the cabinet is stored in a place only members of the research team know of. | |
| Digital research data | |

| In which system will the research data be collected and managed?  X Castor EDC / Research Manager / …. (strongly advised)  ☐ In a (Excel or SPSS) file, saved in a study map on SharePoint Wetenschap, saved with a password and only accessible by the research team.   \| <Systeem, locatie en instelling> \| \| --- \|   ☐ Otherwise: . |
| --- | --- |
| Is digital research data (also) stored on a hospital network drive?    X Yes, on network drive Sharedir Data (K:) > SHAREDIR > TZO private.  However, only coded data will be saved on this drive. *Ga naar vraag 5.1.6*  ☐ No, digital research data is saved on <locatie>. *Ga naar vraag 5.2.1* |
| Which persons have access to this location on the network drive?  This should be at least two people because of access to the data in case of absence, illness, leaving the institution, etc.    Only research staff from the burn center have access to this TZO private file. The only map with patient specific data (Eligibility Files) is secured by a password only known by C.H. van der Vlies (principal investigator), R.F.C. Salemans (coordinating investigator), M.E. van Baar (researcher), D. van Uden (researcher), S.M. Thambithurai (researcher), M.C. Heijblom (research nurse), H.W.C. Hofland (nurse scientist), M.A.C.M. de Craen (research nurse).  Similar process is in place in the joining center Red Cross Hospital Beverwijk and Martini Hospital Groningen of which the secured password is only known by the allocated research team members. |

| How is it ensured that no one other than the research team has access to this location on the network drive? (multiple answers possible)    X Only the research team has access to the location on the network drive  ☐ The study folder is protected with a password  X Study documents are protected with a password   \| <klik hier om uw tekst in te voeren> \| \| --- \|   ☐ Other: . |
| --- | --- |
| Data processing |
| Will research data be anonymized or coded?    ☐ Anonymized *Ga naar vraag 5.2.4*  X Coded *Ga naar vraag 5.2.2* |
| How are data coded?  In Maasstad Burn Center Rotterdam patients will be coded as follows: LK_R01, LK_R02, etc.  Red Cross Burn Center Beverwijk: LK_B01, LK_B02, etc.  Martini Burn Center Groningen: LK_G01, LK_G02, etc. |

| Where is the participant identification code list (key between coded and patient traceable data) stored?    X In the study folder on the departmental disk, protected with a password and only accessible to the study team. The key does not leave Maasstad ziekenhuis.  ☐ External. For external storage of the key including access privileges, permission is requested from the patient in the patient information form (PIF). <Instelling en afdeling extern> |
| --- |
| By whom will the required data be extracted from the electronic patient file and anonymized or coded?  ☐ Treating healthcare professional  X Helpers (under supervision of treating physician): for Rotterdam: Roos Salemans, Denise van Uden, Marscha Heijblom, Helma Hofland, Monique de Craen    ☐ Department of business information (BI) |
| Data sharing |
| Will research data be shared with third parties during study?    ☐ Yes, anonymized *ga door naar vraag 5.3.2*  ☐ Yes, coded *ga door naar vraag 5.3.2*  X No *ga door naar vraag 5.4* |

| To whom is the data provided / who uses the data for scientific research?    ☐ Institutions/researchers in the Netherlands: <Noteer hier de naam en locatie van de instelling(en).>  ☐ Institutions/researchers in the European Union (EU): <Noteer hier de naam en locatie van de instelling(en).>  ☐ Institutions/researchers outside the EU: <Noteer hier de naam en locatie van de instelling(en).> | |
| --- | --- |
|  | |
| How long will data be saved? | |
| Data will be kept for 15 years after which it will be destroyed. | |
| VALORIZATION AND PUBLICATION | |
| Valorization | |
| The results of this study will help answer the worldwide ongoing debate about appropriate timing of surgery in adult patients with deep partial-thickness burns. A strength of this study is that it examines both clinical and patient-reported outcomes. Therefore, this multicenter study would be an excellent addition to the current mostly dated and inconclusive literature.  In addition, the information gathered by this study will be used in the future to develop a decision aid that will assist physicians and patients in making a more informed decision about the timing of surgery or conservative therapy. | |
| Publication | |
| This study’s methodology, processes, and results will be published in scientific journals, and the findings will be presented at (international) conferences, allowing the information generated by this study to be broadly disseminated. | |
| REFERENCES | |
| 1. Hebron, C., et al., *Implementation of the World Health Organization Global Burn Registry: Lessons Learned.* Ann Glob Health, 2022. **88**(1): p. 34. 2. Van Yperen, D.T., et al., *Epidemiology of burn patients admitted in the Netherlands: a nationwide registry study investigating incidence rates and hospital admission from 2014 to 2018.* Eur J Trauma Emerg Surg, 2022. **48**(3): p. 2029-2038. 3. Karim, A.S., K. Shaum, and A.L.F. Gibson, *Indeterminate-Depth Burn Injury-Exploring the Uncertainty.* J Surg Res, 2020. **245**: p. 183-197. | |
| 4. | Anna Miroshnychenko, K.K., Bram Rochwerg, Sophocles Voineskos, *Comparison of early surgical intervention to delayed surgical intervention for treatment of thermal burns in adults: A systematic review and meta-analysis.* Burns Open, 2021. **5**: p. 67-77 |
| 5. | Herndon, D.N., et al., *A comparison of conservative versus early excision. Therapies in severely burned patients.* Ann Surg, 1989. **209**(5): p. 547-52; discussion 552-3. |
| 6. | Mohammadi, A.A., et al., *Early excision and skin grafting versus delayed skin grafting in deep hand burns (a randomised clinical controlled trial).* Burns, 2011. **37**(1): p. 36-41. |
| 7. | Ayaz, M., et al., *Effects of Early Versus Delayed Excision and Grafting on Restoring the Functionality of Deep Burn-Injured Hands: A Double-Blind, Randomized Parallel Clinical Trial.* J Burn Care Res, 2019. **40**(4): p. 451-456. |
| 8. | Engrav, L.H., et al., *Early excision and grafting vs. nonoperative treatment of burns of indeterminant depth: a randomized prospective study.* J Trauma, 1983. **23**(11): p. 1001-4. |
